# Enhancing Endocannabinoid Control of Stress with Cannabidiol

**DOI:** 10.3390/jcm10245852

**Published:** 2021-12-14

**Authors:** Jeremy D. Henson, Luis Vitetta, Michelle Quezada, Sean Hall

**Affiliations:** 1 Prince of Wales Clinical School, University of NSW, Sydney, NSW 2052, Australia; luis.vitetta@sydney.edu.au; 2 Medlab Clinical Ltd., Sydney, NSW 2015, Australia; michelle_quezada@medlab.co (M.Q.); sean_hall@medlab.co (S.H.); 3 Faculty of Medicine and Health, The University of Sydney, Sydney, NSW 2006, Australia

**Keywords:** stress, hypothalamus–pituitary–adrenal axis, endocannabinoid, N-arachidonylethanolamine, AEA, cannabidiol, CBD

## Abstract

The stress response is a well-defined physiological function activated frequently by life events. However, sometimes the stress response can be inappropriate, excessive, or prolonged; in which case, it can hinder rather than help in coping with the stressor, impair normal functioning, and increase the risk of somatic and mental health disorders. There is a need for a more effective and safe pharmacological treatment that can dampen maladaptive stress responses. The endocannabinoid system is one of the main regulators of the stress response. A basal endocannabinoid tone inhibits the stress response, modulation of this tone permits/curtails an active stress response, and chronic deficiency in the endocannabinoid tone is associated with the pathological complications of chronic stress. Cannabidiol is a safe exogenous cannabinoid enhancer of the endocannabinoid system that could be a useful treatment for stress. There have been seven double-blind placebo controlled clinical trials of CBD for stress on a combined total of 232 participants and one partially controlled study on 120 participants. All showed that CBD was effective in significantly reducing the stress response and was non-inferior to pharmaceutical comparators, when included. The clinical trial results are supported by the established mechanisms of action of CBD (including increased N-arachidonylethanolamine levels) and extensive real-world and preclinical evidence of the effectiveness of CBD for treating stress.

## 1. The Stress Response

### 1.1. Physiology and Pathology of the Stress Response

The stress response has two arms: (i) the hypothalamus–pituitary–adrenal (HPA) axis secretion of cortisol and (ii) the sympathetic nervous system (SNS) release of noradrenaline and adrenaline. The HPA axis and SNS are interconnected and act in parallel to increase the ability to cope with the immediate stress and avoid similar threats in future [1,2,3]. The “fight or flight” reaction is part of the acute stress response that mobilizes glucose and increases blood flow to the muscles. Mentally, attention to the threat is increased by facilitating arousal, increasing focus on the threat, and increasing awake time. Distraction from the threat is reduced by increasing analgesia and inhibiting immediately unhelpful behaviors/processes such as feeding, reproduction, and growth. Memory consolidation is also facilitated so that the threat can be identified in future [2,3,4,5]. Some aspects of the stress response may not be appropriate for modern-day stressors that do not require increased physical activity to be dealt with. 

Fear, anxiety, and depressive behaviors are normal physiological aspects of the stress response, can be beneficial, and are not pathological states [4,6]. Fear is an unpleasant emotional response to an immediate threat that can enhance mental functions, including attention. Anxiety is also an unpleasant emotional response that can enhance mental functions; however, it is in response to a future (not immediate) threat. Anxiety creates hypervigilance in anticipation of the future threat to help individuals prepare for dangers and facilitates memory consolidation for alerting to repeated occurrences [6]. Depressive behaviors, such as anhedonia, may help reduce distraction from the threat. Fear, anxiety, and depressive behaviors are only diagnosed as mental disorders when they are both (i) persistent or are elicited by non-threatening stimuli and (ii) hinder the ability to function normally [6].

Stress can cause considerable harm. While the acute stress response improves survival, if it is excessive or chronic, stress can be a causative factor for or exacerbate numerous somatic and mental illnesses. In order to prioritize relevant activities for dealing with the immediate threat, the stress response changes key processes in the cardiorespiratory, gastrointestinal, and immune systems; growth, sex, and thyroid hormone axes; and executive, cognitive, fear, anger, reward, and wake–sleep centers of the brain, as well as increasing inflammatory cytokine levels [2]. If excessive or persistent, these changes can lead to substantial pathology. For example, stress can promote (i) inflammatory diseases such as asthma, eczema or urticaria, rheumatoid arthritis, ulcerative colitis, and sickness syndrome; (ii) pain disorders such as headaches and abdominal, pelvic, and lower back pain; (iii) gastrointestinal dysfunction such as diarrhea, constipation, and peptic ulcers; (iv) mental illnesses such as anxiety, depression, psychosis, cognitive dysfunction, and insomnia; (v) metabolic diseases such as diabetes mellitus, hypercholesterolemia, visceral obesity, and sarcopenia; (vi) ischemic heart disease; (vii) neurodegenerative diseases; and (viii) osteopenia and osteoporosis [2,3,4,7,8,9,10]. Chronic or repeated stress is especially harmful and is a major factor in precipitating or exacerbating mental illnesses such as anxiety disorders and depression [9]. In some systems, the appropriate intensity and duration of the stress response can improve functioning and health; however, an excessive or prolonged stress response reduces it. For example, acute stress can improve immune system functioning, whereas excessive or chronic stress suppresses the immune response to bacterial and viral infections, vaccinations, and cancer [2,11,12]. Similarly, acute stress increases analgesia, whereas chronic stress can cause hyperalgesia.

Habituation or adaptation of the stress response is important for minimizing the adverse effects from a repeated stress response [13,14]. With habituation, the stress response decreases in intensity with repeated events of the same stressor. For example, when a person starts living next to a railway line, the sound of a train hurtling past may induce a substantial stress response; however, the intensity dwindles over time. This habituation is important for reducing what can be thought of as the wear and tear load on the mind and body from an ongoing stress response [15,16].

### 1.2. Measures of the Stress Response

The stress response can be measured clinically by assessing either or both of its two arms. The HPA axis is usually measured by assessing cortisol levels, which are increased with acute stress. Cortisol levels in healthy individuals have a diurnal pattern, peaking in the early morning and having their low point early in the night-time. Chronic stress can cause a flattening of the diurnal pattern, lowering the early morning peak and elevating the low night-time levels [9]. The sympathetic nervous system (SNS) arm of the stress response causes an increase in circulating catecholamines, increased heart rate, decreased heart rate variability (especially decreased high-frequency heart rate variability), and raised salivary alpha-amylase [9,17,18,19,20]. Symptoms of the stress response should also be assessed using validated questionnaires such as the Subclinical Stress Symptom Questionnaire-25 (SSQ-25) [21] or the Perceived Stress Scale-10 (PSS-10) [22]. For specifically measuring occupational stress, the Psychological Strain Questionnaire (PSQ) can be used, which is part of the larger Occupational Stress Inventory—Revised (OSI-R) [23]. Some validated anxiety questionnaires also assess stress, e.g., the Depression Anxiety Stress Scales (DASS) [24].

### 1.3. Impact and Current Treatment Options for Stress

Stress can be defined as an event that is perceived to threaten homeostasis [2]. The common types of life stressors and their average stress response intensity with respect to causing illness are listed in the Social Readjustment Rating Scale (SRRS; also known as the Holmes and Rahe Stress Scale) [25]. While occupational issues do not rate as highly as changes in personal relationships, health problems, and legal issues, occupational stress provides an insight into the substantial impact stress has on the individual and the economy. In Australia, in 2015, 91% of workers’ compensation claims involving a mental health condition were linked to occupational stress. The claims caused by occupational stress, comprised 5.5% of all workers’ compensation claims and 16% of all payouts and caused 17% of all time off work due to compensation claims [26]. Apart from increasing the number of sick and personal leave days, occupational stress also reduces productivity and work quality when employees are at work by causing an increased rate of accidents, poor communication, increased conflict, reduced job satisfaction and morale, reduced client satisfaction, and increased staff turnover [12,14].

There is a need for interventions in the stress response to prevent health disorders from eventuating and to reduce unhelpful symptoms. Treatments to reduce stress responses include pharmacological and psychological approaches. Many of the medications that are used for stress relief are addictive, and all have the potential for serious adverse reactions and drug interactions. In addition, many can take several weeks before their effects are experienced, and medications that only inhibit one arm of the stress response can be undermined by a compensatory increase in the other [1]. Prescription medications used for stress and its manifestations include beta blockers [1], benzodiazepines [27,28], selective serotonin reuptake inhibitors [27], and bupropion [29]. Psychological methods for treating occupational stress include meditation, relaxation, biofeedback, and cognitive and behavioral therapy. Meditation and relaxation techniques, such as thought reduction (mental silence) or progressive muscle relaxation, are used to reduce physiological symptoms of the stress response and, thereby, reduce the reactivity of the individual to occupational stressors [23]. Biofeedback techniques aim to reduce reactivity by training people on how to gain conscious control over stress processes such as heart rate and brain wave patterns. Cognitive behavioral techniques include changing the way the person appraises the stressful situations and an individual’s own perceived ability to deal with the situation, as well as improving coping techniques [14]. When individuals believe that they can manage a stressor, they experience a less intense stress response [23].

## 2. The Endocannabinoid System

The endocannabinoid signaling system is one of the key regulators of the stress response and is important for proper return to the non-stressed state. The endocannabinoid system constrains the magnitude of the stress response, promotes return of the HPA axis to non-stressed levels, and facilitates habituation of the stress response to repeated or ongoing stress. It also directly inhibits stress-associated processes such as fear, anxiety, depressive behaviors, inflammation, and hyperalgesia and promotes behaviors inhibited by the stress response such as feeding and sleep. The effect of the endocannabinoid system can be summarized as promoting a cool, calm, collected, fat, and happy state [13]. Furthermore, resilience to stress-related disease and dysfunction may depend on the satisfactory functioning of the endocannabinoid system [4,30,31,32,33].

**Figure 1:**
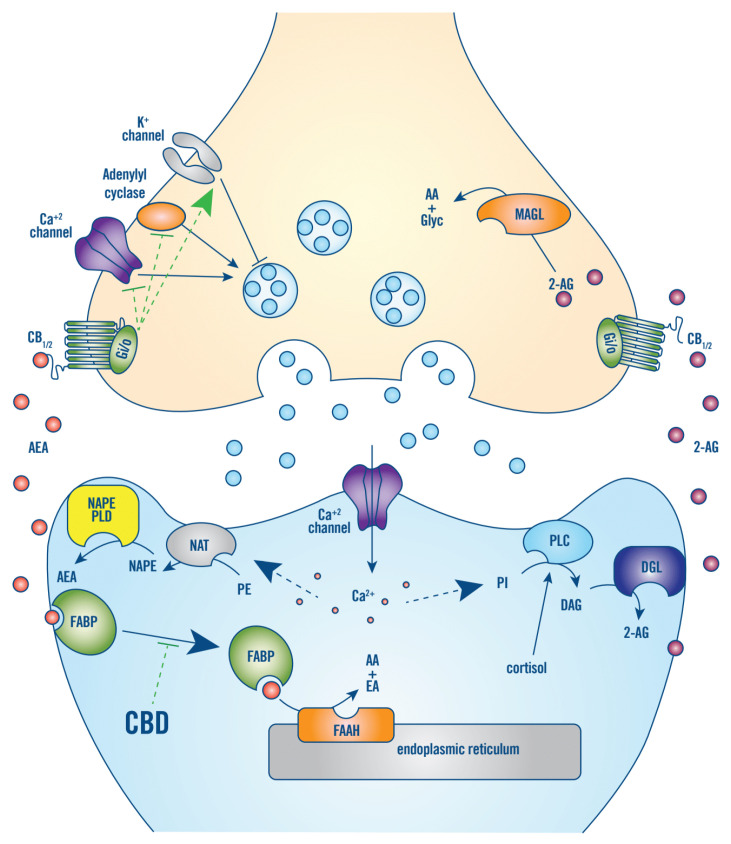
CB_1/2_ signaling in the nervous system. The two main endocannabinoids, AEA and 2-AG, are synthesized in response to neuronal depolarization and/or Ca^+2^ influx, via cleavage of membrane phospholipids, such as phosphatidylethanolamine (PE), in the postsynaptic membrane. For AEA, Ca^+2^-dependent N-acyltransferase (NAT) first produces N-arachidonoyl PE (NAPE), which is then hydrolyzed by phospholipase D (NAPE-PLD). For 2-AG, Ca^+2^ influx and/or cortisol stimulates phospholipase C (PLC), which hydrolyzes phosphatidylinositol (PI) into diacylglycerol (DAG), which is hydrolyzed by diacylglycerol lipase (DGL). AEA and 2-AG feedback in a retrograde manner to CB_1/2_ receptors on presynaptic terminals. CB_1/2_ are coupled to G_i/o_-proteins that function to inhibit adenylyl cyclase and voltage-gated calcium channels and activate potassium channels, thus, suppressing afferent neurotransmitter release. In the brain, CB_1/2_ signaling has a basal tone that depends on AEA production in response to neuronal activity. This tone can be rapidly reduced, by increased hydrolyzation of AEA to arachidonic acid (AA) and ethanolamine (EA) by FAAH that is in the post-synaptic endoplasmic reticulum. The main action of CBD is to competitively inhibit binding of AEA to its aqueous transporter, fatty acid binding protein (FABP), thereby inhibiting the degradation of AEA by FAAH and increasing CB_1/2_ receptor signaling tone. Moreover, 2-AG is not degraded by FAAH but by monoacylglycerol lipase (MAGL), which is located near CB_1/2_ on the pre-synaptic membrane [15,39].

The endocannabinoid system is an evolutionarily conserved signaling system that was discovered in the 1990s after the identification of the primary targets of tetrahydrocannabinol (THC) and the cannabinoid receptors type 1 (CB_1_) and type 2 (CB_2_). CB_1_ are one of the most abundant G-protein coupled receptors in the central nervous system and are located on presynaptic terminals where they suppress neurotransmitter release, mostly on excitatory glutamatergic neurons and inhibitory gamma-aminobutyric acid (GABA)ergic interneurons, and to a lesser extent serotonergic, noradrenergic, and dopaminergic neurons [15]. CB_2_ are mostly located in immune cells (include microglia) and modulate immune cell migration and cytokine release. There is also evidence that CB_2_ are present on neurons in stress-associated areas of the brain and regulate the release of GABA, dopamine, and glutamate [34,35,36]. The two main endogenous cannabinoids (endocannabinoids), N-arachidonylethanolamine (anandamide; AEA) and 2-arachidonoylglycerol (2-AG), are synthesized in response to neuronal depolarization and/or Ca^+2^ influx, via cleavage of membrane phospholipids. In the nervous system, this occurs in the postsynaptic membrane and the endocannabinoids feedback in a retrograde manner to CB_1/2_ on presynaptic terminals, thus inhibiting afferent neurotransmitter release (Figure 1) [15,37]. AEA and 2-AG are hydrophobic, and it is not known how they cross the aqueous synaptic space; however, AEA produced by microglia cells may be transported to presynaptic CB_1_ via microvesicles [38]. AEA also binds peroxisome proliferator-activated receptor-γ and transient receptor potential vanilloid member 1 [13].

In the brain, CB_1/2_ signaling has a basal tone that depends on AEA production in response to neuronal activity. This tone can be rapidly reduced by increased degradation of AEA by fatty acid amide hydrolase (FAAH) that is located in post-synaptic endoplasmic reticulum (Figure 1) [15,39]. CB_1/2_ signaling is increased by increasing the levels of 2-AG production by phospholipase C [40]. Phospholipase C is activated by Ca^+2^ influx secondary to neuronal depolarization and cortisol [3]. Furthermore, 2-AG is not degraded by FAAH but by monoacylglycerol lipase (MAGL), which is located near CB_1/2_ on the pre-synaptic membrane (Figure 1) [15]. In addition to their respective primary degradative enzymes, AEA and 2-AG can also be oxygenated by cyclooxygenase 2 (COX-2) to form bioactive prostaglandin derivatives [15].

## 3. Regulation of the Stress Response by the Endocannabinoid System

CB_1_ expression is especially high in the cortico-limbic brain regions associated with the stress response, and CB_1_ signaling constrains the stress response centrally via both its HPA axis and sympathetic nervous system arms [9,30,31,32]. Both CB_1_ and CB_2_ signaling are also involved in mediating the central and peripheral manifestations of the stress response [13]. Most research and understanding of the role of the endocannabinoid system in stress has come from animal (rodent) studies that employ unconditioned stressors and then monitor changes in the balance between exploration and avoidance behaviors [31,32]. Human studies correlating circulating AEA and 2-AG levels with aspects of the stress response and studies investigating the stress response effects of CB_1_ inhibition (by rimonabant) have supported that the animal model data can be extrapolated to humans [15].

**Figure 2:**
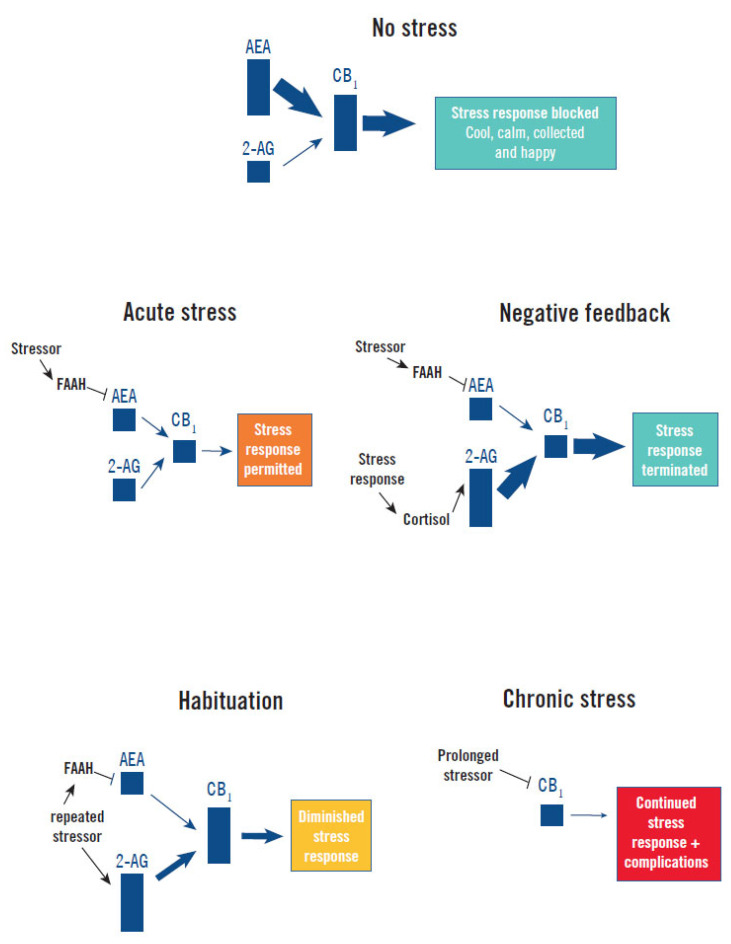
Endocannabinoid regulation of the stress response. Without a stressor (No Stress) basal AEA tone maintains CB_1_ signaling constraint of the stress response and a cool, calm, collected, and happy state. Acute presentation of a stressor (Acute Stress) elevates FAAH hydrolysis of AEA, reducing CB_1_ signaling and permitting activation of the stress response [13,15,32,39]. Secretion of cortisol by the stress response provides Negative Feedback by increasing 2-AG production, which increases CB_1_ signaling and terminates the stress response [15]. Repeated presentation of the same stressor progressively increases 2-AG levels, possibly by reduced MAGL expression and degradation of 2-AG, which causes progressively higher CB_1_ signaling and Habituation to the stress response [4,15]. Chronic Stress causes a downregulation of CB_1_ that impairs feedback inhibition and facilitates persistence of the stress response and high cortisol levels, which precipitates or exacerbates illness (complications) [5,15,40].

Although CB_1_ signaling is known to inhibit noradrenalin release by the SNS [9], more is known about how it regulates the HPA axis. There is tonic inhibition of the HPA axis’s stress response by CB_1_ signaling, which can be modified in the following ways (Figure 2):

1. Stress response induction and maintenance: Acute exposure to stress rapidly increases corticotropin-releasing hormone signaling in limbic structures, which increases the enzymatic activity of FAAH, resulting in a rapid decrease of the inhibitory tone of AEA (and CB_1_ signaling) on the HPA axis [13,15,32]. This mechanism continues to maintain low AEA levels as long as the stressor remains [13].
2. Stress response termination: Increased levels of cortisol following induction of the stress response stimulates production of 2-AG in the hypothalamus and other stress centers of the brain, increasing CB_1_ signaling. This applies negative-feedback inhibition of the HPA axis and can facilitate termination of the stress response [15].
3. Habituation of the stress response: Upon repeated presentation of the same stressor, 2-AG levels are progressively enhanced within forebrain stress centers, increasing CB_1_ signaling and HPA axis habituation [4,15]. This increase in 2-AG may be due to a reduction in MAGL expression.
4. Chronic dysfunction: Exposure to chronic stress causes decreased CB_1_ expression in the stress centers of the brain due to epigenetic changes [15,40]. This results in less feedback inhibition on the HPA axis and contributes to continued high levels of cortisol following chronic stress [5].

In addition to regulating the stress response, decreased levels of CB_1_ and CB_2_ signaling also have direct effects on the manifestations and complications of the stress response, which makes CB_1_ and CB_2_ signaling a good target for reducing many undesirable effects of acute and chronic stress. CB_1_ signaling on forebrain glutamatergic neurons reduces anxiety specifically under stressful or aversive situations [41,42,43]. Chronic stress induces neuroinflammation and activates microglia (the brain-resident macrophages), which can facilitate anxiety and depressive behaviors and contribute to the development of affective disorders. Both CB_1_ and CB_2_ signaling can prevent the activation of microglia, cytokine signaling, and stress-induced recruitment of monocytes to the brain’s neurovascular space, which may help constrain neuroinflammation [4,9,15,36]. CB_1_ and possibly CB_2_ are present in the enteric nervous system, and CB_2_ is also expressed on immune and epithelial cells of the gastrointestinal tract (GIT). CB_1_ and CB_2_ signaling opposes the increased GIT pain sensitivity, motility, inflammation, immune activation, and permeability caused by acute and/or chronic stress [13,40]. Chronic impairments in CB_1_ and CB_2_ signaling may also directly contribute to long-term complications of chronic stress, such as learning and memory deficits, changes in coping behaviors, post-traumatic stress disorder, anxiety disorders, depression, psychosis, and pain syndromes [15,16,44]. For example, CB_1_ signaling extinguishes fear and can prevent persistence of aversive memories, which if impaired may promote post-traumatic stress disorder (PTSD) and phobias [3,4,44]. Administration of medicines such as FAAH inhibitors or endocannabinoid reuptake inhibitors have been shown to ward off the development of adverse effects of chronic stress [3,4,15,44].

## 4. Cannabidiol (CBD) as a Treatment for Stress

Cannabis has been used medicinally for thousands of years in various societies around the world to reduce the physiological and psychological consequences of stress and fear [13,45]. Of the two main components of cannabis, cannabidiol (CBD) and tetrahydrocannabinol (THC), CBD appears to be the component responsible for these effects. Although THC is a weak partial agonist of CB_1_ and CB_2_, as far as the stress response is concerned, THC appears to act as a competitive inhibitor of AEA and 2-AG at CB_1_, and THC increases basal- and stress-induced glucocorticoids [13]. CBD products (edibles, tinctures, and vapes) are commonly used around the world to treat stress, as well as self-perceived anxiety and insomnia, which often may be symptoms of stress [46,47]. Because CBD acts on several synergistic targets, it may be more effective (as well as safer) for constraining the stress response than molecules designed to target specific endocannabinoid receptors or degradative enzymes [31,32].

### 4.1. Real-World Evidence

In the UK, over 10% of adults have tried CBD [46] and, in the USA, in one month, there were over 6 million internet searchers for CBD [48]. Between 35% and 65% of people using CBD for medicinal purposes in the UK, USA, Denmark, and New Zealand were found to be administering it for stress [46,47], and over 90% reported feeling less stressed with CBD, with no respondents reporting feeling more stressed [46]. For stress and its manifestations of mild anxiety and insomnia, CBD is usually administered at low doses and mostly from online suppliers, with less than 1% being prescribed by a doctor and less than 5% purchased from a pharmacy [46].

### 4.2. Mechanism of Action

CBD increases CB_1_ and CB_2_ signaling by increasing AEA levels. CBD increases AEA levels in rodents (usual animal model for stress) by inhibiting the enzymatic activity of FAAH. In humans, CBD does not enzymatically inhibit FAAH but inhibits AEA degradation by FAAH indirectly—by preventing transport of AEA from the post-synaptic membrane to FAAH, which is mainly located in the post-synaptic endoplasmic reticulum. CBD competitively inhibits AEA binding to fatty acid binding proteins that transport hydrophobic AEA across the aqueous space between the plasma membrane and the endoplasmic reticulum (Figure 1) [39]. CBD treatment has been shown to increase AEA levels in the serum of schizophrenia patients [49].

**Figure 3:**
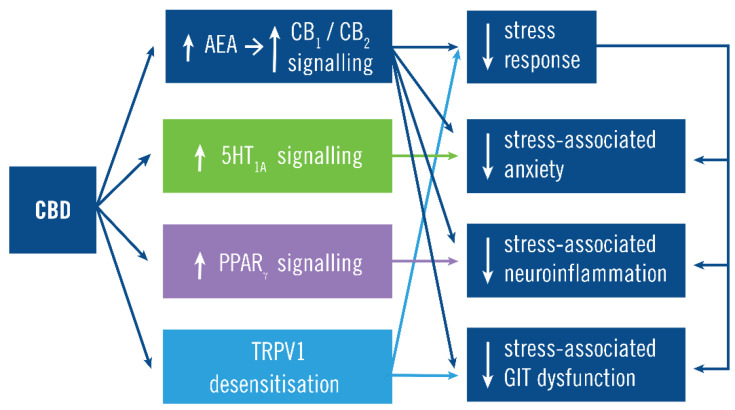
Cannabidiol (CBD) mechanism of action. The main action of CBD is to increase CB_1_ and CB_2_ signaling by preventing N-arachidonylethanolamine (AEA) degradation. This constrains the stress response and its manifestations both via its inhibition of the stress response and (independently) inhibition of some manifestations such as the anxiety, neuroinflammation, and GIT dysfunction. For example, CB_1_ signaling inhibits stress-associated anxiety by both constraining the stress response and by CB_1_ activity on forebrain glutamatergic neurons [41,42,43]. CBD also acts independently of CB_1_/CB_2_ signaling by increasing serotonergic 5HT_1A_ signaling, increasing PPARγ signaling and desensitizing TPRV1, which inhibit stress-associated anxiety, neuroinflammation and GIT dysfunction, respectively.

CBD also acts to reduce stress and its manifestations by non-endocannabinoid receptors (Figure 3). Even at low doses, CBD acts as an agonist at serotoninergic 5-HT_1A_ receptors and blocks stress-induced changes in 5-HT_1A_ receptor gene expression, which reduces anxiety associated with the stress response [50,51,52]. CBD also activates peroxisome proliferator-activated receptor gamma (PPARγ) that reduces neuroinflammation and excitotoxicity that is associated with the stress response [53]. It is possible that CBD could also reduce the stress response via binding the transient receptor potential vanilloid member 1 (TRPV1), because CBD is known to desensitize TRPV1 [54] and TRPV1 is a mediator of the stress response [31,32,55]. The differential expression and differential sensitivity to modulation of CB_1_ on different types of neurons may also be important for the net effect of CBD [56,57,58,59]. CBD can act as a negative allosteric modulator of CB_1_ [60,61] and CB_2_ [62] (in vitro), thereby acting as a non-competitive antagonist of the actions of THC and endogenous CB_1/2_ agonists. Although CBD does antagonize some actions of THC, CBD does not have the same effects as CB_1/2_ antagonists (such as recombinant) [61], and CBD largely regulates the stress response by increasing CB_1/2_ signaling [37,63,64].

### 4.3. Safety, Tolerability, and Pharmacokinetics

Low-dose CBD that appears effective for stress and its manifestations [46] has a good safety and tolerability profile, with few adverse effects [65,66,67,68,69]. Unlike THC, CBD is not psychomimetic and does not cause intoxication, euphoria, addiction, psychomotor impairment, or cognitive impairment [70,71,72]. Importantly, low-dose CBD (less than 150 mg/day) does not cause the hepatocellular injury observed for higher dose CBD (>600 mg/day) [73,74,75,76]. Reviews of 49 clinical trials of CBD, including intravenous, inhalation, and oral routes of administration and oral dose ranges of 10–1500 mg per day, found that CBD was well tolerated with a good safety profile [65,77]. CBD has also been shown to have no potential for abuse or dependence in humans [78,79,80,81]. High-dose CBD has drug–drug interactions with medicines metabolized by the cytochrome P450 pathways [82], and the extent to which this occurs with low-dose CBD is not yet known. There is a possibility of mild drowsiness and fatigue with the low-dose CBD [82].

CBD is currently delivered per-orally (ingested), by absorption across the oral mucous membranes or by inhalation [83,84,85,86,87]. Because CBD is poorly water soluble, ingestion provides poor absorption, and most of the CBD that is absorbed undergoes first-pass metabolism, which results in a bioavailability of only 6% [84,88]. Systemic exposure to CBD is increased four-fold by ingestion with a high-fat meal [86] and five-fold with severe hepatic impairment [89]. The main reason for the large increase in absorption with a high-fat meal is that micelles are naturally formed in the small intestine by the mixing of bile salts with fatty acids from the high-fat meal (or an oil-based CBD formulation), and these micelles carry CBD into intestinal epithelial cells and the portal circulation [90]. First-pass metabolism may be bypassed by the delivery of CBD across the oral mucous membrane; however, many CBD sublingual drops still result in high levels of first-pass metabolites, which indicates that mucous membrane absorption is inefficient [91]. Oral mucous membrane absorption could be improved by mimicking the carriage of CBD in natural intestinal micelles, by formulating CBD in synthetic nano-micelles [87]. Inhaling CBD by smoking or vaping provides the most rapid method of administration, with a time-to-peak-plasma-concentration (T_max_) ≤5 min and a bioavailability of 31% [92]; however, the higher peak level from inhalation is associated with increased adverse effects [91], and the high temperatures involved produce toxic oxidation products [93]. 

The single-dose half-life of CBD is around 3 h; however, CBD accumulates in tissues, including adipose tissues due to its lipophilicity, and after repeated doses, its half-life is 2–5 days [84]. CBD is also binds to proteins and blood cells and has a high apparent volume of distribution of 32 L/kg [88,92]. Low-dose CBD administration provides serum CBD levels in the order of 1–10 ng/mL [83,84,85,87,91]. CBD is mainly metabolized in the liver by CYP3A- and CYP2C-dependent phase I metabolism to its active metabolite 7-OH-CBD, which is then metabolized and excreted in feces and urine after phase II metabolism by uridine 5′-diphospho-glucuronosyltransferase (UGT) enzymes [84,88].

### 4.4. Preclinical Evidence of Efficacy

CBD attenuates the effects of experimentally induced acute and chronic stress in animal models (rodents) by increasing both CB_1_ and CB_2_ signaling and by facilitating 5-HT_1A_ receptor-mediated neurotransmission. Increased CB_1_ and CB_2_ signaling due to FAAH inhibition by CBD attenuated stress-associated anxiety behaviors, prevented the chronic stress-associated decrease in hippocampal neurogenesis, and prevented the persistence of fear [37,64]. The anxiolytic effect of CBD was not seen in unstressed animals, indicating that CBD induced anti-stress rather than anxiolytic effects [64]. Moreover, dependent on CB_1_ and CB_2_ activation, CBD treatment has been shown to prevent the chronic stress-induced decrease in total dendritic length, number of branches, spine density of neurons, and expression of synaptic proteins in the hypothalamus and other limbic structures [63,64]. CBD has also been shown to act in 5-HT_1A_-receptor-dependent ways to reduce stress-associated anxiety behaviors, heart rate, blood pressure, and fear expression. Again, the anxiolytic effects were only seen after stress (acute or chronic) [37,52].

### 4.5. Clinical Evidence of Efficacy

The first clinical evidence that CBD reduces the stress response was from the studies of CBD’s ability to reduce the adverse effects of THC in healthy volunteers [94,95]. THC is known to induce stress in healthy people as demonstrated by an increase in cortisol and transient anxiety-like behavior in people with no anxiety disorders [13]. Karniol et al. reported that a single dose of CBD of 15–60 mg significantly reduced THC-induced anxiety, and CBD doses of 30–60 mg significantly reduced THC-induced tachycardia, which like anxiety is part of the stress response [94]. These results were supported by Zuardi et al. (1982), who showed that a single CBD dose of 1 mg/kg (50–80 mg; average of 67 mg) significantly lowered THC-induced anxiety as measured by the State-Trait Anxiety Inventory (STAI) [95]. In both studies, CBD alone did not change the pulse rate or anxiety levels, indicating that its effects were stress specific [94,95].

CBD has also been shown to reduce stress-response-associated anxiety caused by public speaking and radiological tests. Zuardi et al. (1993) exposed healthy subjects (without any psychiatric diagnosis) to the acutely stressful situation of a simulated public speaking test and measured the anxiety component of the stress response after treatment with placebo, 300 mg CBD, 10 mg diazepam, or 5 mg ipsapirone (5-HT_1A_ agonist). CBD treatment significantly constrained the stress-induced increase in the STAI and Visual Analog Mood Scale (VAMS) measures of anxiety by a similar magnitude to diazepam and ipsapirone, without the sedation associated with diazepam. Again, the actions of CBD were stress specific with no effect seen on anxiety measures before the stress response [27]. The same group confirmed these results in 2017, demonstrating that 300 mg of CBD had comparable efficacy to 1 mg of clonazepam in lowering stress-induced anxiety and heart rate [28]. This time, the healthy participants (no current or prior psychiatric disorders) underwent a test of public speaking and stress-induced anxiety measured with the VAMS showed that CBD had a U-shaped efficacy curve, with less efficacy reported for 100 and 900 mg doses of CBD [28]. Crippa et al. used a different stressor, single positron emission computed tomography (SPECT) scanning, which included intravenous cannula insertion and tracer injection [96]. In healthy subjects with no current, past, or family (immediate family) history of psychiatric disorders, 400 mg CBD (dissolved in corn oil) significantly lowered stress-induced anxiety (as measured by VAMS) relative to placebo [96]; however, this time some mental sedation from CBD was reported. The stressor, SPECT scanning, was also used to demonstrate that the stress-associated anxiolytic action of CBD was associated with reduced blood flow to the cortical limbic and paralimbic brain areas suggesting that the effect of CBD involved these areas of the brain, which are known to be involved in the stress response and its behavioral manifestation of anxiety [96].

CBD may be helpful in not only reducing acute stress-associated anxiety but also normalizing abnormal stress responses. Appiah-Kusi et al. studied the effects of 600 mg CBD on Trier Social Stress Test (TSST)–induced anxiety and cortisol level changes in participants who had no history of mental health disorder; however, these participants were judged at high risk of developing psychosis based on the Comprehensive Assessment of At-Risk Mental States (CAARMS) questionnaire. These participants were found to have decreased rather than increased cortisol levels in response to acute stress. CBD treatment attenuated the abnormal cortisol response and reduced the acute stress-associated increase in anxiety, as measured by the STAI [8].

Another behavioral manifestation of stress is fear. CB_1_ signaling is important for extinguishing fear after stress, and lack of fear extinction following stress is thought to be a major contributor to the development of fear and anxiety disorders [3,4,37,44]. Das et al. studied the persistence of fear and aversive memories due to electric shock in healthy volunteers and found that 32 mg CBD was effective in enhancing fear and aversive memory extinction [97].

Crippa et al. (2021) studied the effectiveness of 150 mg CBD twice a day on 120 healthcare workers with burnout syndrome [98]. Burnout syndrome is a manifestation of chronic stress that has been described and is only defined for workplace (occupational) stress and is not considered a mental health disorder [99]. Burnout syndrome is characterized by (i) feelings of energy depletion or exhaustion; (ii) increased mental distance from one’s job or feelings of negativism or cynicism related to one’s job; and (iii) a sense of ineffectiveness and lack of accomplishment [99,100]. This study was not fully blinded or placebo controlled for ethical reasons; however, it did show that by 14 days, CBD treatment provided significant decreases in the emotional exhaustion from burnout syndrome and associated symptoms of anxiety and depression and had a medium effect size for treating burnout syndrome [98].

Although the ability to reduce anxiety caused by anxiety disorders is not necessarily predictive of ability to treat physiological stress-induced anxiety, CBD has been studied in people with anxiety disorders. There have been two clinical trials of CBD in social anxiety disorder (SAD). The first study used the Simulation Public Speaking Test in individuals diagnosed with SAD, to show that a 400–600 mg single dose of CBD significantly reduced subjective symptoms of anxiety, cognitive impairment, and performance discomfort, relative to the placebo group [77,101]. A recent double-blind study on teenagers (18–19 years old) with SAD and avoidant personality disorder showed that 300 mg/day CBD for four weeks significantly decreased anxiety compared to placebo [102]. Low-dose CBD has been studied in a recent clinical trial of CBD for patients with any anxiety disorder, which found 25 mg/day CBD to be effective [75].

## 5. Conclusions

In summary, the stress response is in need of a safe, rapid, effective treatment to prevent associated morbidities and economic losses. There is substantial clinical evidence that CBD safely and effectively constrains the stress response. There have been seven double-blind placebo controlled clinical trials of CBD for stress [8,27,28,94,95,96,97] on a combined total of 232 participants and one partially controlled study on 120 participants. All showed that CBD was effective in significantly reducing the stress response and its manifestations (anxiety, fear, depression, and burnout). Two clinical trials [27,28] included a comparator (benzodiazepines and/or 5HT_1A_ agonists) arm, and both showed that the CBD effect was non-inferior to that of the pharmaceutical drug. The clinical trial results are supported by the common (unregulated) use of CBD by over 10% of the population, of which 1/3–2/3 use it to relieve stress and 90% find effective, and by the established mechanism of action and extensive preclinical evidence of the effectiveness of CBD for treating stress. Maladaptive stress responses and the endocannabinoid system as a therapeutic target both deserve more attention from clinicians and researchers, and CBD may be a good solution to both.

## Search Strategy and Selection Criteria

References for this review were identified through searches of PubMed for articles published from January 2000 to June 2021, by use of the terms “stress”, “endocannabinoid”, and “cannabidiol” and through searches in the authors’ personal files. Articles resulting from these searches and relevant references cited in those articles were reviewed.
